# The Expression Pattern of Hypoxia-Related Genes Predicts the Prognosis and Mediates Drug Resistance in Colorectal Cancer

**DOI:** 10.3389/fcell.2022.814621

**Published:** 2022-01-27

**Authors:** Ye Yuan, Lulu Tan, Liping Wang, Danyi Zou, Jia Liu, Xiaohuan Lu, Daan Fu, Guobin Wang, Lin Wang, Zheng Wang

**Affiliations:** ^1^ Research Center for Tissue Engineering and Regenerative Medicine, Union Hospital, Tongji Medical College, Huazhong University of Science and Technology, Wuhan, China; ^2^ Department of Gastrointestinal Surgery, Union Hospital, Tongji Medical College, Huazhong University of Science and Technology, Wuhan, China; ^3^ Department of Clinical Laboratory, Union Hospital, Tongji Medical College, Huazhong University of Science and Technology, Wuhan, China

**Keywords:** hypoxia, colorectal cancer, prognosis, drug resistance, gene signature, tumor microenvironment

## Abstract

**Background:** Colorectal cancer (CRC) is one of the leading causes of cancer-related deaths worldwide. However, due to the heterogeneity of CRC, the clinical therapy outcomes differ among patients. There is a need to identify predictive biomarkers to efficiently facilitate CRC treatment and prognosis.

**Methods:** The expression profiles from Gene Expression Omnibus (GEO) database were used to identify cancer hallmarks associated with CRC outcomes. An accurate gene signature based on the prognosis related cancer hallmarks was further constructed.

**Results:** Hypoxia was identified to be the primary factor that could influence CRC outcomes. Sixteen hypoxia-related genes were selected to construct a risk gene signature (HGS) associated with individuals’ prognosis, which was validated in three independent cohorts. Further, stromal and immune cells in tumor microenvironment (TME) were found to be associated with hypoxia. Finally, among the 16 hypoxia-related genes, six genes (*DCBLD2*, *PLEC*, *S100A11*, *PLAT*, *PPAP2B* and *LAMC2*) were identified as the most attributable ones to drug resistance.

**Conclusion:** HGS can accurately predict CRC prognosis. The expression of the drug resistance-related genes is critical in CRC treatment decision-making.

## Introduction

Colorectal cancer (CRC) is the second leading cause of cancer-related death. In 2020, there were approximately 1 million CRC-related deaths worldwide.^1^ Since the majority of CRC cases are diagnosed at an advanced stage, CRC patients’ prognosis is often poor. Moreover, given CRC’s high heterogeneity in molecular genetics and histopathology, the standard chemo/radio therapy may not be effective across all individuals. Thus, prognosis prediction is of great importance in clinical practice to improve individual treatment. In the past decades, tumor, node, and metastasis (TNM) staging system has been widely used to predict treatment outcomes of cancers. However, the accuracy of this system is non-satisfactory (Kuipers et al., 2015). In recent years, advances in genomic research such as next-generation sequencing have revolutionized cancer treatment. Genomic data is currently being utilized for cancer screening and accurate monitoring of drug responses (Frampton et al., 2013; Tsimberidou et al., 2014; Meric-Bernstam et al., 2015).

Metastasis and uncontrolled growth of solid tumors often require new blood vessels. The newly-formed microvasculature is highly abnormal in structure and function, leading to a hypoxia tumor microenvironment (TME) (Jain, 2005), known to promote cancer progression, angiogenesis, and metastasis (Yotnda et al., 2010; Chang et al., 2011; Shao et al., 2018). Besides, hypoxia is thought to mediate chemo/radio resistance through multiple mechanisms (Yoshiba et al., 2009; Xu et al., 2019; Yue et al., 2019; Lu et al., 2020) and become a high-priority target in cancer therapy. Several bioreductive prodrugs that are activated enzymatically from hypoxia induction have been developed (Brown, 1993; Wilson and Hay, 2011). However, given that physiological hypoxia occurs in normal tissues, the off-target effects of the prodrugs cannot be ignored (Wilson and Hay, 2011). Also, the variations in tumor hypoxia hinder optimal application of bioreductive prodrugs (Hoogsteen et al., 2009). Thus, tools for stratification of hypoxia situation of CRC patients are crucial for effective cancer therapy.

In the present study, we confirmed that hypoxia was of dominant prognostic significance for CRC. And we established a hypoxia-related gene signature (HGS) and validated the prognosis prediction model in three independent cohorts. Finally, we explored the correlation of hypoxia with stromal and immune cells, and identified its role in drug resistance.

## Materials and Methods

### Data Collection and Preprocessing

Expression profile of CRC tissues in GSE39582, GSE38832, and GSE161158 datasets were downloaded from the GEO database (Supplementary Table S1).^2^ TCGA-COAD transcriptome cohort data and clinical data, containing 279 CRC samples, were downloaded from the UCSC Xena website (Supplementary Table S1).^3^ GSE39582 dataset, consisting of 518 CRC samples, was used as the training cohort to construct the prognosis prediction model and external validation was performed in the other three independent cohorts, including TCGA, GSE38832, and GSE161158. All raw data were normalized and standardized by using the R software package.

### Single Sample Gene Set Enrichment Analysis

R package “GSVA” was used to perform single sample gene set enrichment analysis (ssGSEA). The ssGSEA was applied to explore the enrichment of tumor-related pathways and immune cell infiltration in the GSE39582 database. Tumor-related data sets were obtained from hallmark gene sets in the MSigDB database (Supplementary Table S2).^4^ The characteristic gene set containing 28 immune cell types was downloaded from a recent publication (Charoentong et al., 2017).

### Weighted Gene Co-Expression Networks Analysis

The weighted gene co-expression networks analysis (WGCNA) were constructed using the top 25% most differently expressed genes in the GSE39582 dataset. Among all the soft threshold values, we chose the β value with the highest mean connectivity (*β* = 4). The minimum number of genes was set as 30 for the high reliability of the results. For further quantification of hypoxia related genes and modules, genes with *p* value of less than .001 were retained for subsequent analysis.

### Gene Set Enrichment Analysis

The function of the hypoxia-related genes was explored using gene set enrichment analysis (GSEA). In the training cohort and all validation cohorts, we separately created a chip expression profile and a sample data file and then imported them into GSEA software. *p* < .05 and FDR *p* < .25 were considered to be significant.

### Establishment and Validation of a Colorectal Cancer Prognostic Predictive Signature

The univariate Cox regression analysis was conducted to identify recurrence free survival (RFS) and overall survival (OS) related cancer hallmarks. To select hypoxia related genes associated with prognosis, Lasso penalized Cox regression analysis was applied. Finally, the LASSO Cox regression model was employed to identify hypoxia highly correlated genes and construct the prognostic HGS. For each patient, the coefficients of Logistic Regression were used to calculate the HGS score. The formula is: HGS score = ∑ (coefficient × mRNA expression).

### Construction of Nomogram for Colorectal Cancer Prognosis Prediction

Hypoxia score and relevant clinical parameters were used to construct a nomogram, using the survival and the “rms” package of R. The nomogram was constructed to estimate 1-, 3-, and 5-year survival probabilities. The calibration curve and C index were used to evaluate the performance of the model.

### Stromal and Immune Cells Infiltration

We used xCell to estimate the cellular composition of stromal cells and immune cells in the tumor in the GSE39582 dataset. Immune and stromal cell scores for each sample were calculated. The CIBERSORTx online website was used to evaluate the infiltration of 22 immune cells in each sample.^5^ The absolute abundance of eight immune cell populations of tissue infiltrating immune cells was calculated by R package “MCPcount.”

### Drug Sensitivity Analysis

The GSCALite database is used for drug sensitivity analysis.^6^ The relationship between the expression levels of 16 target genes and drug sensitivity was calculated by Spearman correlation analysis.

### Cell Viability and Gene Expression Assay

Human colorectal cancer cells (HCT116) and their corresponding oxaliplatin-resistant cells (HCT116/L-OHP) were incubated in RPMI1640 containing 10% fetal bovine serum (FBS), supplemented with penicillin (100 units/ml) and streptomycin (100 μg/ml) (Liu et al., 2022). For cell viability assay, HCT116 and HCT116/L-OHP cells (6 × 10^3^ cells per well) were seeded in 96-well plates overnight. Then, the cells were exposed to different concentrations of oxaliplatin for 48 h. The cell viability was evaluated using CCK-8 reagent (MedChemExpress). For gene expression assay, we extracted the total mRNA of HCT116 and HCT116/L-OHP cells and reversely transcribed them to cDNA. We performed Real-time quantitative PCR (qPCR) using AceQ qPCR SYBR Green Master Mix (Vazyme). ABI Stepone Real-Time PCR System (Applied Biosystems) was used to measure the fluorescence. The primers were listed in Supplementary Table S3.

### Statistical Analysis

Data was analyzed using the R software. For forest plots, the hazard ratio (HR) was generated by univariate Cox or multivariate Cox proportional hazard regression. Kaplan-Meier approach was used for the survival analysis. Differences between groups were assessed using the Wilcoxon test. Statistically significant differences were showed as follow: *p < .05; **p < .01; ***p < .001; NS, not significant.

## Results

### Hypoxia Identified as a Primary Risk Factor Related to Colorectal Cancer Prognosis

**FIGURE 1:**
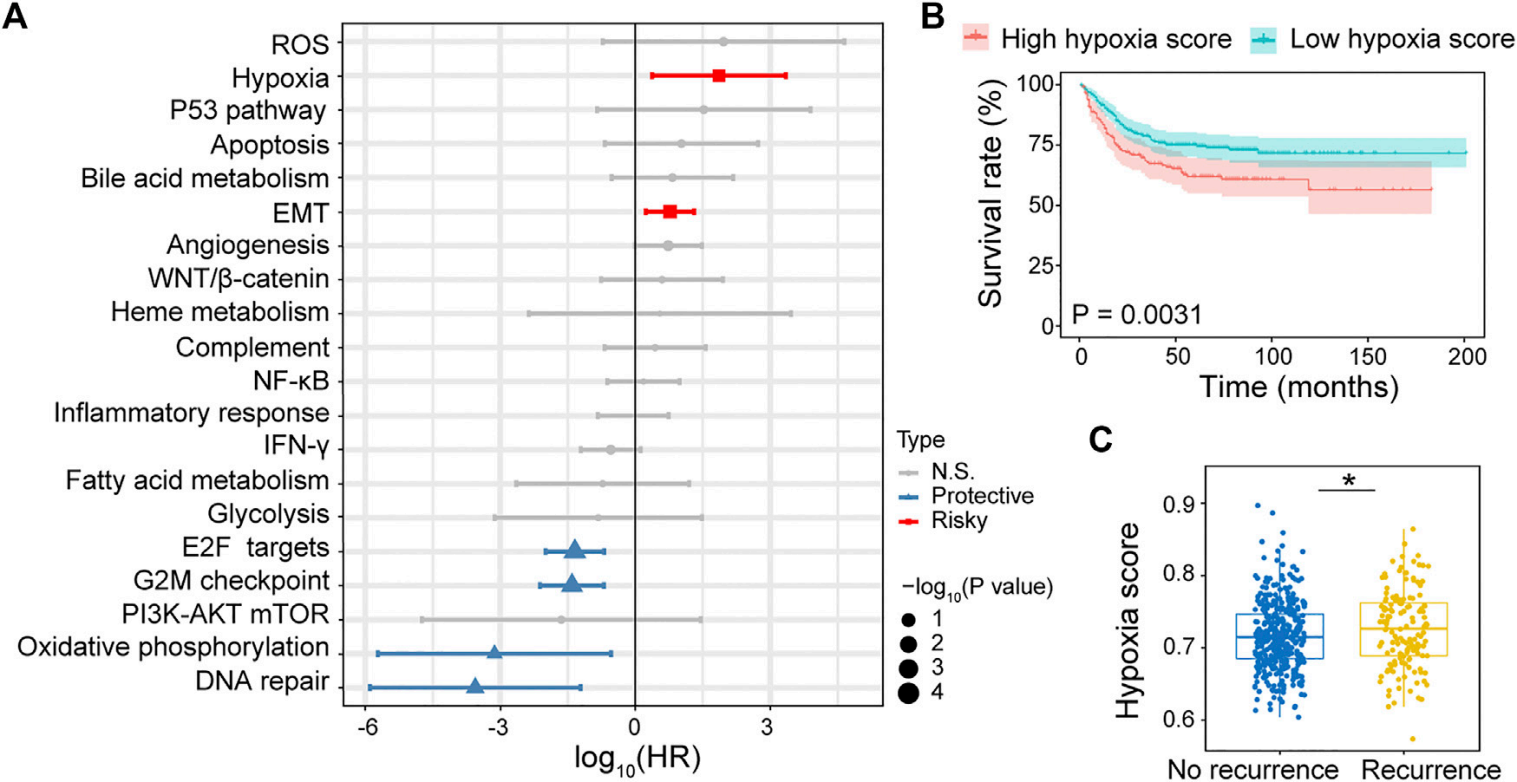
Identifying hypoxia associated with CRC recurrence free survival (RFS). **(A)** Forest plot of hazard ratio (HR) for 20 prognostic cancer hallmarks. **(B)** Kaplan–Meier RFS curves for patients with high hypoxia and low hypoxia scores. **(C)** Comparison of hypoxia scores in recurrence and no recurrence patients.

The RNA-sequencing data of 518 CRC cancer samples obtained from GSE39582 dataset was evaluated using ssGSEA. The association between cancer hallmarks and CRC prognosis was evaluated using Cox regression analysis. We found that hypoxia (HR: 72; 95% CI: 2.4–2,225; *p* = .01) and epithelial-mesenchymal transition (EMT) (HR: 5.9; 95% CI: 1.7–20; *p* = .005) were strongly associated with poor RFS (Figure 1A), whereas the expression of p53 pathway (HR: 209; 95% CI: 1.5–29,514; *p* = .03), hypoxia (HR: 61; 95% CI: 2.9–1,281; *p* = .007), angiogenesis (HR: 5; 95% CI: 1.1–23; *p* = .04), and EMT (HR: 3.3; 95% CI: 1.1–10; *p* = .03) were significantly associated with shorter OS (Supplementary Figure S1A). Overall, hypoxia was the most significant risk factor associated with both poor RFS and OS. Then, the CRC patients were divided into high and low hypoxia score groups using the cutoff selected by X-title program. Compared with low hypoxia score, high hypoxia score was associated with poor RFS and OS (Figure 1B; Supplementary Figure S1B). Meanwhile, hypoxia score in recurrence patients was significantly higher than that in no recurrence patients (Figure 1C; Supplementary Figure S1C). These results reveal that hypoxia is the primary risk factor associated with CRC prognosis.

### Construction of Hypoxia-Related Gene Signature

**FIGURE 2:**
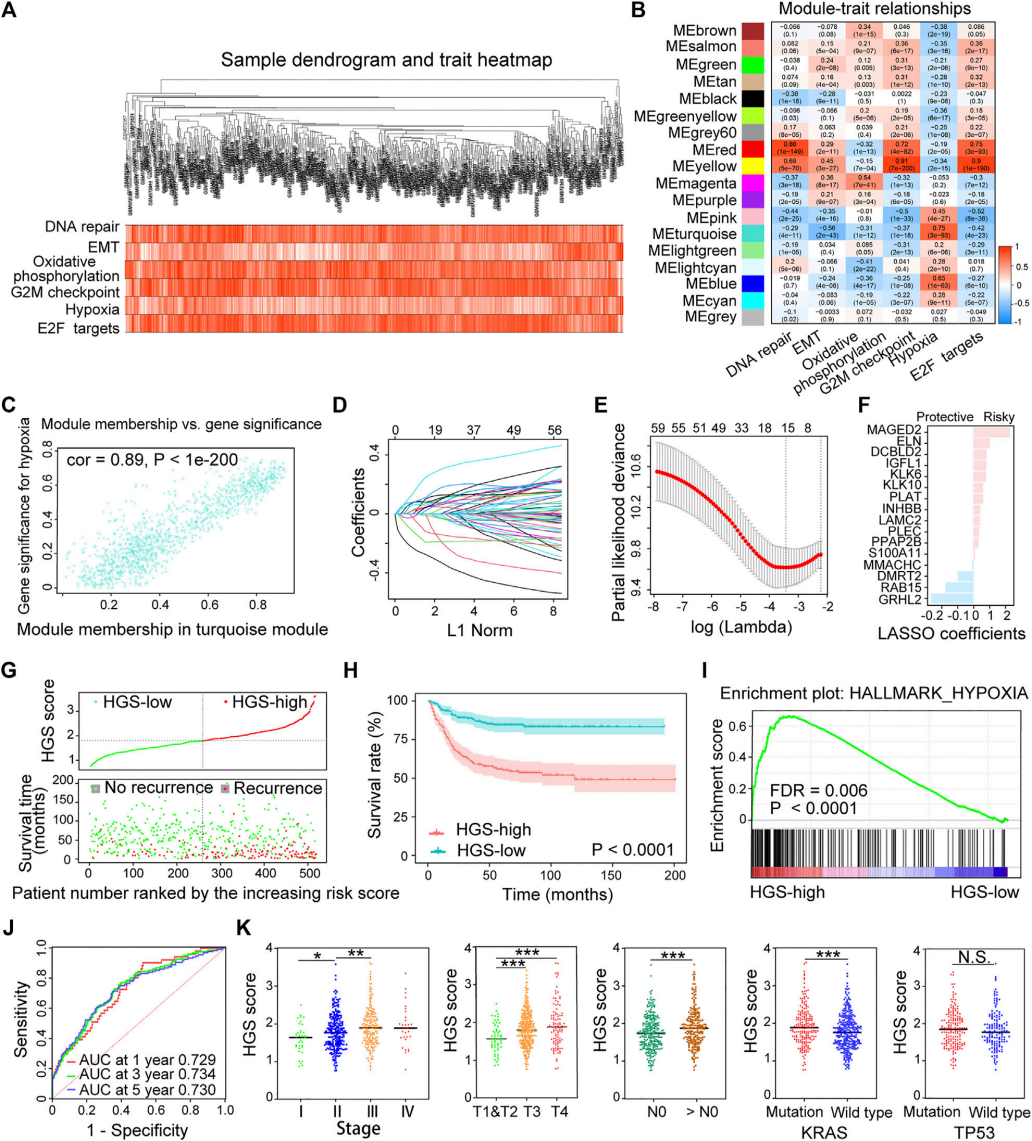
Establishment and validation of HGS in the training cohort. **(A)** Clustering dendrogram of 518 samples from WGCNA. **(B)** Heatmap of the correlation between the modules and cancer hallmarks. **(C)** Correlation between turquoise module and hypoxia. **(D)** LASSO coefficient profiles of the hypoxia-related prognostic differential expressed genes. **(E)** 10-fold cross-validation for penalty parameter λ selection in the LASSO model. **(F)** LASSO coefficients of the 16 hypoxia-related genes. **(G)** The distribution of HGS score, patients’ status and RFS time. **(H)** Kaplan–Meier RFS curves for patients in HGS-high and HGS-low groups. **(I)** GSEA of hypoxia pathway in HGS-high and HGS-low groups. **(J)** Time-dependent ROC curves at 1, 3 and 5 years. **(K)** Correlation of HGS with clinicopathological characteristics.

WGCNA was conducted by comparing the co-expression patterns between hypoxia scores and whole-transcriptome profiling data (Figure 2A). The optimal soft threshold was set to four to construct a scale free network (Supplementary Figure S2). A total of 18 modules were identified (Supplementary Figure S3). The turquoise module strongly associated with hypoxia was selected for further analysis (Figures 2B,C). LASSO Cox regression analysis was applied to select the most useful prognostic markers within the module. Sixteen genes (*MAGED2*, *ELN*, *DCBLD2*, *IGFL1*, *KLK6*, *KLK10*, *PLAT*, *INHBB*, *LAMC2*, *PLEC*, *PPAP2B*, *S100A11*, *MMACHC*, *DMRT2*, *RAB15* and *GRHL2*) were identified and used to construct the optimal HGS (Figures 2D,E). Among them, *MMACHC*, *DMRT2*, *RAB15* and *GRHL2* were associated with better CRC prognosis, whereas the rest were associated with poor prognosis (Figure 2F).

HGS score of each patient was calculated based on the expression levels of the 16 genes. Taking the median HGS score as the cutoff, all patients were divided into HGS-high or HGS-low groups. Survival analysis showed that HGS-high was associated with CRC recurrence (Figure 2G), which was consistent with Kaplan-Meier analysis results (*p* < .0001) (Figure 2H). GSEA revealed that the expression of the HGS-high group genes was associated with hypoxia induction (*p* < .0001) (Figure 2I). The areas under the receiver operating characteristic (ROC) for 1-, 3-, and 5-year RFS of the HGS were 0.729, 0.734, and 0.730, respectively (Figure 2J). Further analysis revealed that high HGS score was related to advanced clinical stages and worse pathological grades as well as *KRAS* gene mutations (Figure 2K). The newly constructed prognostic model shows good predictive performance.

### Validation of Hypoxia-Related Gene Signature

**FIGURE 3:**
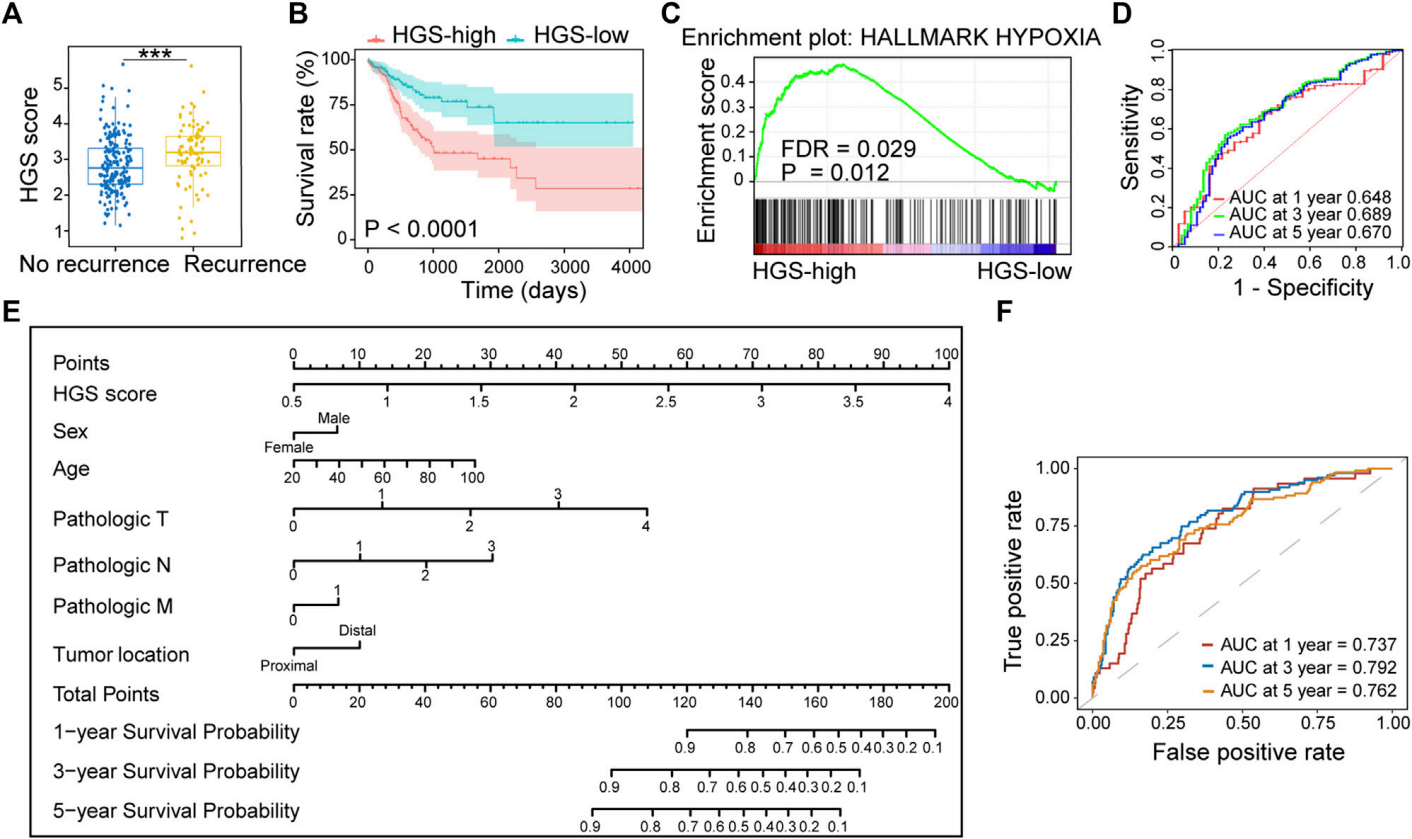
Validation of HGS in TCGA. **(A)** Comparison of HGS scores in recurrence and no recurrence patients. **(B)** Kaplan–Meier RFS curves for patients in HGS-high and HGS-low groups. **(C)** GSEA of hypoxia pathway in HGS-high and HGS-low groups. **(D)** Time-dependent ROC curves at 1, 3 and 5 years. **(E)** Nomogram developed based on HGS and clinicopathological characteristics. **(F)** Time-dependent ROC curves at 1, 3 and 5 years.

The prognostic value of HGS was validated using three independent cohorts (TCGA, GSE38832 and GSE161158). We found that the patients with worse prognosis exerted higher HGS scores (Figure 3A; Supplementary Figures S4A, S5A, S6A). The HGS-high group also displayed shorter RFS (*p* < .0001) (Figure 3B; Supplementary Figures S5B, S6B). GSEA revealed that genes in HGS-high group were enriched in hallmarks of hypoxia (*p* = .009) (Figure 3C; Supplementary Figure S5C). Multivariate Cox regression analysis revealed that TNM stage and HGS score were independent risk factors for RFS (Supplementary Figures S4B, S6C). Furthermore, higher HGS score was associated with lymph node metastasis and advanced TNM stage (Supplementary Figure S4C). The area under the ROC curves for 1-, 3-, and 5-year RFS of HGS were 0.648, 0.689, and 0.670, respectively (Figure 3D).

A nomogram for predicting individual RFS probability of CRC patients was developed by combining HGS and clinicopathological characteristics (sex, age, pathologic T, pathologic N, pathologic M, and tumor location) of the patients (Figure 3E). The areas under ROC for 1-, 3-, and 5-year RFS of the nomogram were 0.737, 0.792, and 0.762, respectively (Figure 3F). Compared with other models, the nomogram showed the most powerful ability for RFS prediction. The probabilities for 1-, 3-, and 5-year survival showed excellent agreement between prediction by nomogram and observed values (Supplementary Figure S7). Taken together, the newly constructed nomogram can accurately predict CRC prognosis.

### Mechanism of Hypoxia in Tumor Microenvironment

**FIGURE 4:**
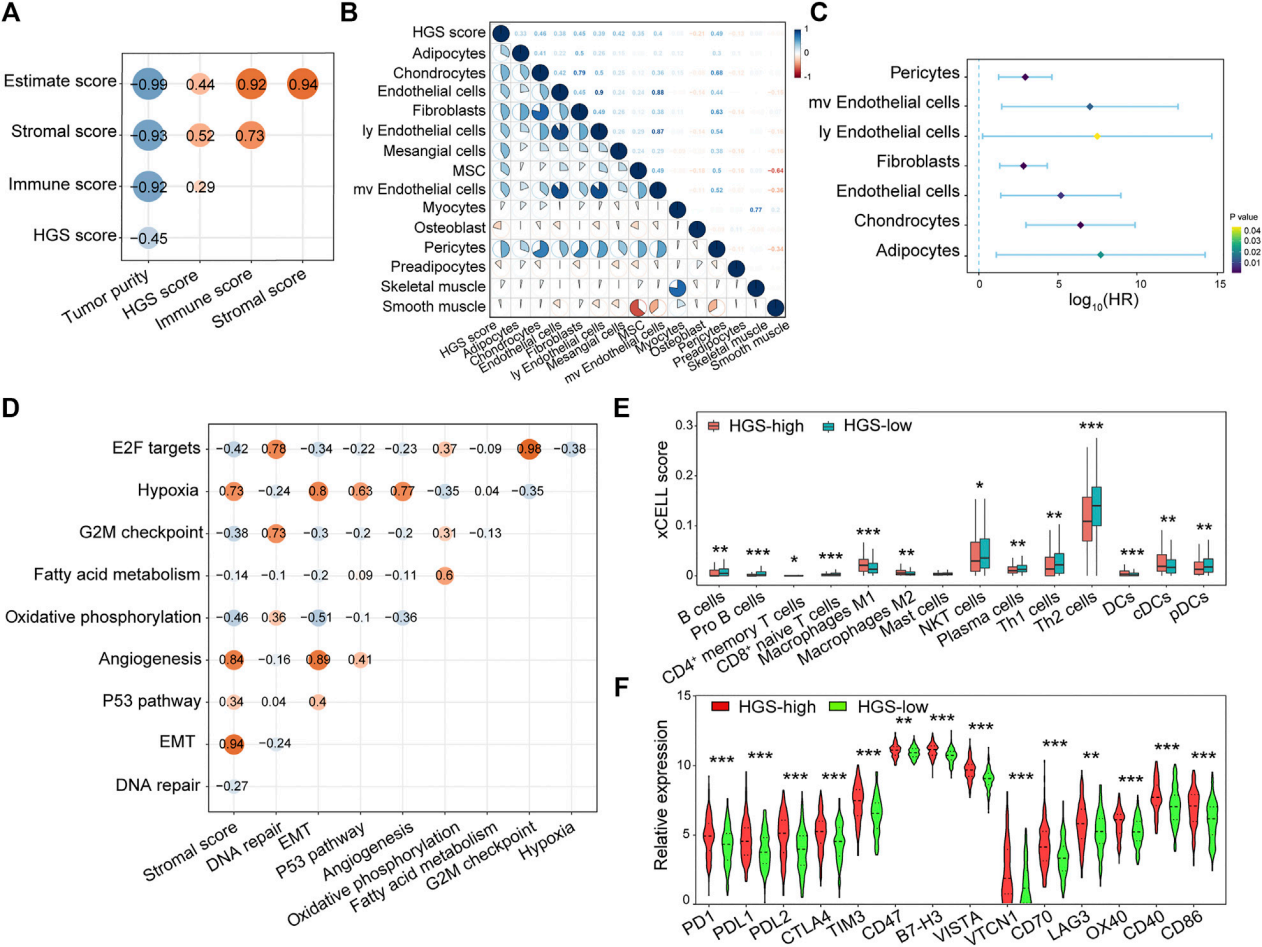
The correlation between HGS and immune cells, stromal cells infiltration. **(A)** Spearman correlation analysis between HGS score and immune cells, stromal cells infiltration. **(B)** The correlation between HGS score and various stromal cells. **(C)** Forest plot of HR for various prognostic stromal cells. **(D)** The correlation between hypoxia and cancer hallmarks. **(E)** The infiltration level of 14 immune cells. **(F)** The expression pattern of immune checkpoint-related and immune active genes.

To clarify the potential role of hypoxia in TME, we carried out correlation analysis between HGS and immune and stromal cells. The results showed that both Immune score and Stromal score were positively correlated with HGS score (Figure 4A). A correlation matrix between HGS and stromal cells revealed that pericytes, mv endothelial cells, ly endothelial cells, fibroblasts, endothelial cells, chondrocytes, and adipocytes were significantly more abundant in HGS-high group (Figure 4B; Supplementary Figure S8), and they were associated with worse RFS (Figure 4C). Moreover, EMT was positively correlated with Stromal score (Figure 4D), suggesting its potential role mediated by stromal cells in tumor progression.

Interestingly, 13 immune cells were found to be associated with hypoxia in CRC. Among them, B cells, pro B cells, CD4^+^ memory T cells, CD8^+^ naïve T cells, NKT cells, plasma cells, Th1 cells, Th2 cells, and pDCs were significantly higher in HGS-low group individuals, whereas M1 macrophages, M2 macrophages, DCs and cDCs were significantly higher in HGS-high group individuals (Figure 4E; Supplementary Figure S9). To evaluate the immune response between the two groups, the expression of *PD1*, *PDL1*, *PDL2*, *CTLA4*, *TIM3*, *CD47*, *B7-H3*, *VISTA*, *VTCN1*, *CD70*, and *LAG3* immune checkpoint-related genes as well as *OX40*, *CD40*, and *CD86* immune active genes were analyzed. The Wilcoxon test revealed that all sets of genes were overexpressed in HGS-high group (Figure 4F). Therefore, hypoxia may be an efficient target to improve immunotherapy.

### The Correlation Between Hypoxia-Related Gene Signature and Drug Resistance

**FIGURE 5:**
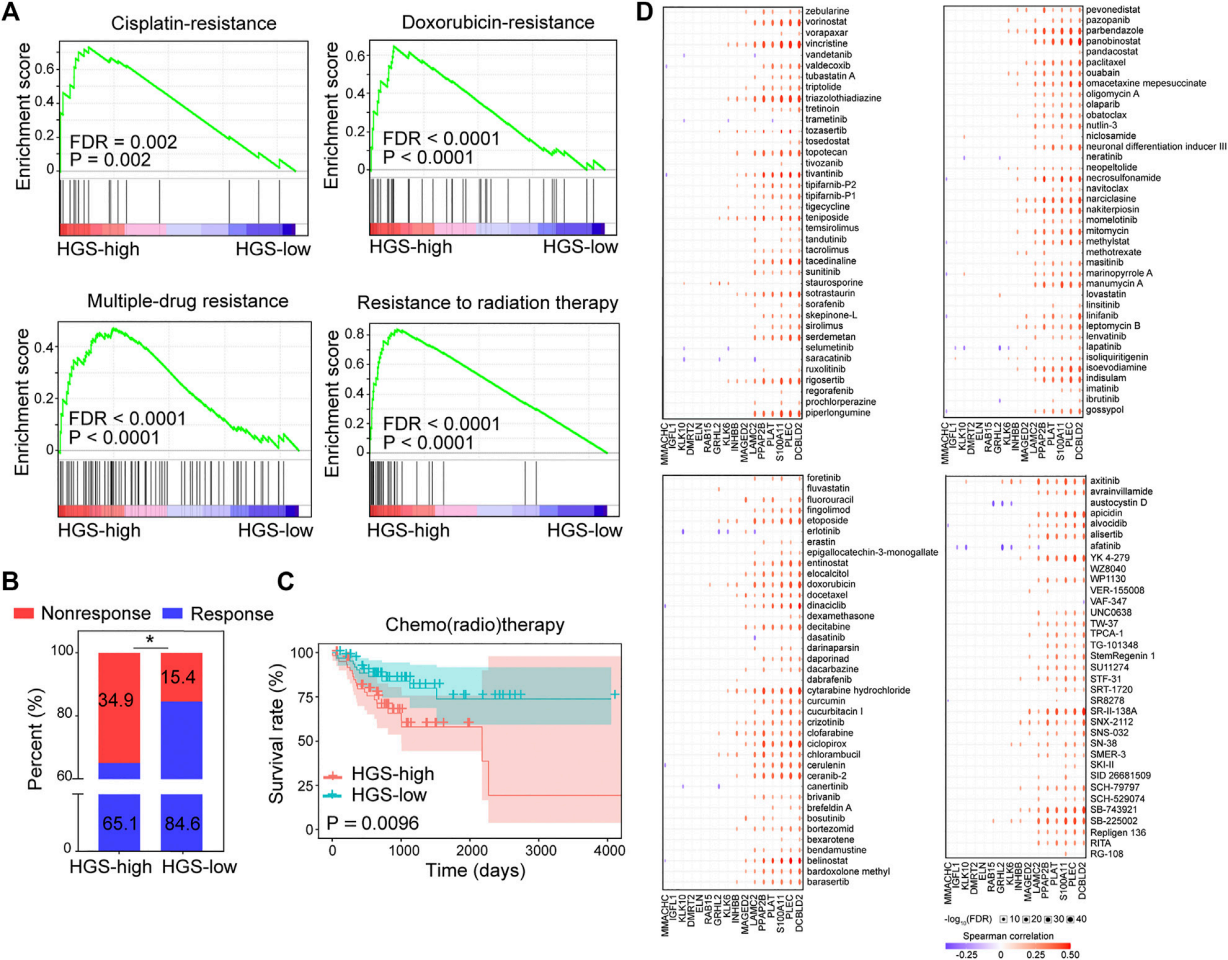
The correlation between HGS and drug resistance. **(A)** GSEA of cisplatin-resistance, doxorubicin-resistance, multidrug-resistance, and resistance to radiation therapy pathways in HGS-high and HGS-low groups. **(B)** Comparison of CRC chemotherapy treatment response between HGS-high and HGS-low groups. **(C)** Kaplan–Meier RFS curves for patients in HGS-high and HGS-low groups. **(D)** Spearman correlation analysis between the 16 hypoxia genes and 151 chemo drugs.

GSEA of gene sets related to chemo/radio response revealed that cisplatin-resistance, doxorubicin-resistance, multidrug-resistance, and radiation-resistance gene sets were over-expressed in HGS-high group (Figure 5A). Moreover, a substantial proportion of patients in HGS-high group responded poorly to chemo/radio therapy (Figure 5B). Survival analysis revealed that HGS-high group was strongly associated with worse RFS after chemo/radio therapy (Figure 5C). Spearman correlation of the 16 hypoxia genes and 151 chemo drugs in the Genomics of Drug Sensitivity in Cancer database revealed that the expression of *DCBLD2*, *PLEC*, *S100A11*, *PLAT*, *PPAP2B* and *LAMC2* was associated with resistance to most chemotherapies (Figure 5D). Furthermore, qPCR tests revealed that these six genes were overexpressed in drug-resistant HCT116/L-OHP cells (Supplementary Figure S10). These findings further conform that HGS is a useful tool in predicting drug resistance and with potential clinical value.

## Discussion

Hypoxia participates in the initiation, progression, metastasis, and drug resistance of CRC (Li et al., 2017; Chen et al., 2020; Qureshi-Baig et al., 2020; Yang et al., 2020). In the present study, we found that hypoxia was the primary risk factor associated with both poor OS and shorter RFS of CRC patients. A CRC prognosis prediction model based on 16 hypoxia-related genes was constructed and validated in three independent CRC cohorts. Besides, an accurate CRC prognosis prediction nomogram combining the expression profile of hypoxia-related genes and clinicopathological characteristics of CRC patients was also constructed.

Apart from tumor cells, TME comprises of stromal and immune cells embedded in the extracellular matrices. While adapting to hypoxia, stromal cells and immune cells play critical roles in tumor progression. In this study, we found that stromal cells were more abundant in TME of patients in the HGS-high group, including mesenchymal stem cells (MSCs), fibroblasts, endothelial cells, and adipocytes among others. Experimental evidence revealed that hypoxia promoted recruitment of MSCs to primary breast tumors as well as tumor metastasis to lymph nodes and lungs (Chaturvedi et al., 2013). Hypoxia-inducible factor 1α (HIF-1α)-induced extracellular matrix remodeling by fibroblasts promoted the invasion and metastasis of cancer cells (Gilkes et al., 2013). Meanwhile, endothelial cells remained to be a non-proliferating state under normal condition. However, they became activated by hypoxia to initiate angiogenesis (Nagl et al., 2020). Endothelial cells could also promote tumor metastasis by directly allowing tumor cells into blood vessels or through EMT process (Joseph et al., 2018; Sobierajska et al., 2020). Besides, adipocytes promoted breast cancer progression by secreting metabolic substrates, such as leptin, which stimulated hypoxia at tumor sites (Chu et al., 2019).

Furthermore, our findings showed distinct lymphocytes infiltration between HGS-high and HGS-low groups. Th1 and Th2 cells, differentiation of naive progenitor cells, are commonly studied subtypes of CD4^+^ T cells. Th1 cells activates CD8^+^ T cells and NKT cells by expressing IFN-γ. Th2 cells exerts their anti-tumor property by recruiting eosinophils through the expression of IL-4 and IL-13 (Kim and Cantor, 2014). We found that hypoxia decreased infiltration of Th1 cells and Th2 cells to tumor sites. It was reported that hypoxia influenced the subtype differentiation of CD4^+^ T cells. HIF-1α reportedly decreased differentiation to Th1 cells and further suppressed production of IFN-γ from these cells (Shehade et al., 2015). Besides, CD4^+^ T lymphocytes particularly Th1 subtypes participated in vessel normalization, suggesting their ability in abrogating hypoxia (Tian et al., 2017). CD8^+^ T cells are the primary adaptive immunocytes mediating tumor cytotoxicity. In the present study, we found that hypoxia was closely related to decreased CD8^+^ naïve T cells infiltration. As suggested by previous studies, hypoxia decreased CD8^+^ T cells recruitment to tumor site, and even caused T cells apoptosis by inducing the production of reactive oxygen species (ROS) (Hildeman et al., 1999; Lukashev et al., 2006; Hasmim et al., 2013). NKT cells are a distinct lineage of T cells with both T cell receptors and NK cell receptors, thus exerting wide ranges of immune effector properties. Our results showed hypoxia could decrease the infiltration of NKT cells, consistent with the reported finding that HIF-2α inhibited activation of NKT cells after ischemia-reperfusion injury (Zhang et al., 2016). Nevertheless, the role of hypoxia on the function of NKT cells is not well understood. Apart from the typical residence in the lymphoid tissues, B cells have also been found in the TME. Together with T cells, B cells could mediate immune responses (Nelson, 2010). We found hypoxia suppressed infiltration of B cells in CRC micro-environment. Consistent with our results, Lee et al. (2016) reported that inhibition of HIF-1α increased the infiltration of B-cells in pancreatic ductal adenocarcinoma tissues.

DCs are classified into two major lineages, myeloid or conventional DCs (cDCs), which are functioned in antigen processing and presenting, and plasmacytoid DCs (pDCs), which produce massive IFNs (Wu and Shortman, 2005). We found that high hypoxia promoted infiltration of total DCs and cDCs, whereas pDCs were found more in HGS-low group. How hypoxia participates in activation of DCs is however not well understood. HIF-1α reportedly promoted migration of DCs via the chemokine receptor CCR7, and stimulated their maturation by enhancing the expression of costimulatory molecule CD86 (Kohler et al., 2012), consistent with the elevation of costimulatory molecules in Figure 7B. Even so, little is known about the relationship between hypoxia and the lineages of DCs. Available evidence showed significantly decreased circulating pDCs because of hypoxia while the proinflammatory factors were overexpressed (Yilmaz et al., 2016). Given that DCs play pivotal role in antigen presentation, there would be crosstalk between DCs and other immunocytes involving hypoxia. In a visceral leishmaniasis infection model, excess secretion of HIF-1α from DCs inhibited the production of IL-12, decreasing the number of Th1 subtypes (Hammami et al., 2018).

Tumor-associated macrophages (TAMs) are the primary tumor-infiltrating cell types. M1 and M2 are the major functional subtypes of macrophages (Yunna et al., 2020). In this study, we found that hypoxia significantly increased the infiltration of both M1 and M2 macrophages. Th1 cytokines induced HIF-1α overexpression in macrophages, leading to M1 macrophages polarization, whereas HIF-2α induced by Th2 cytokines promoted M2 response (Takeda et al., 2010). In response to hypoxia, both M1 and M2 macrophages led to immunosuppression. Specifically, macrophages increased the secretion of NO via the HIF-1α signaling pathway, suppressing the function of T cells (Doedens et al., 2010). HIF-2α counteracted the effects of HIF-1α by reducing arginine required for NO synthesis. However, reduced arginine impairs the functioning of T cells in return (Rodriguez et al., 2007; Doedens et al., 2010).

Our analyses further revealed that hypoxia increases the expression of several immune checkpoints including *PD1*, *PDL1*, *PDL2*, *CTLA4*, *TIM3*, *CD47*, *B7-H3*, *VISTA*, *VTCN1*, *CD70*, and *LAG3*. In related studies, hypoxia significantly increased the expression of PD-L1 on DCs, macrophages and tumor cells, and induced the apoptosis of T cells (Noman et al., 2014). Besides, HIF-1α induced expression of CD 47 on cancer cells to avoid phagocytosis by microphages (Zhang et al., 2015). These findings underline the therapeutic potential of targeting hypoxia in CRC treatment.

Other molecular pathways also involve in hypoxia related poor prognosis and drug resistance. We found that hypoxia showed positive correlation with angiogenesis, EMT and p53 pathway, and showed negative correlation with E2F targets, oxidative phosphorylation, DNA repair and G2/M checkpoint. Uncontrolled proliferating tumors need much blood supply and develop hypoxic microenvironment. Hypoxia stimulates angiogenesis, resulting in the formation of immature, abnormal, and leaky neovessels. However, these neovessels are often insufficient to meet the elevated nutrients and oxygen demand of more cancer cells, which in turn aggravate hypoxia. As a result, the highly hypoxic and disordered vascular microenvironment promotes cancer cells’ invasion and distant metastasis (Carmeliet and Jain, 2011). EMT is essential for cancer cell mobility and metastasis. Hypoxia influences the invasive behavior of cancer cells through EMT, which is characterized by the downregulation of epithelial-associated genes and upregulation of mesenchymal-like genes (Hsu et al., 2000; Kim et al., 2002; Manotham et al., 2004). Meanwhile, hypoxia activates the expression of *Snail* and *Slug* to promote EMT (Muz et al., 2015). Interestingly, these two genes were reported to promote resistance to radiation and chemotherapy in ovarian cancer (Marie-Egyptienne et al., 2013). Tumor-associated mutant p53 proteins promote tumor progression through multiple mechanisms (Donehower et al., 2019; Levine, 2019; Zhang et al., 2020). Hypoxia was reported to increase mutant p53 protein levels in cancer cells (Mantovani et al., 2019; Zhang et al., 2020). In return, mutant p53 promoted angiogenesis, helping the adaption of cancer cells to hypoxia (Zhang et al., 2021). E2F family involves in numerous pathways, like cell cycle regulation, DNA damage repair and apoptosis (Pediconi et al., 2003). It was reported that hypoxia repressed BRCA1 expression by compromising the function of E2F1, resulting in tumor progression (Bindra et al., 2005). Oxidative phosphorylation requires constant O_2_ for ATP generation. Under hypoxic conditions, oxidative phosphorylation slows down and glycolysis increases to reduce O_2_ consumption. It was reported that HIF-1 reprogram glucose metabolism and promote mitochondrial autophagy to switch cancer cells from oxidative to glycolytic metabolism (Semenza, 2013). Hypoxia affects DNA repair through multiple mechanisms, including suppressing homology-directed repair, base excision and repair mismatch repair (Kaplan and Glazer, 2020). It is a key microenvironmental factor contributable to genetic instability (Reynolds et al., 1996). The genetic instability could lead to the activation of oncogenes or inactivation of suppressor genes, resulting in the growth and metastasis of cancer cells (Bristow and Hill, 2008). Cell cycle checkpoints are activated upon the generation of DNA damage. The reoxygenation after acute hypoxia generates DNA damage, thus eliciting G2 checkpoint. Chronic hypoxia (hours to days) may not activate checkpoints and thus lead to genetic instability (Bristow and Hill, 2008). As a result, the increased genetic instability enhances the proliferation of cancer cells.

Hypoxia has been shown to promote acquired resistance to conventional chemo/radio therapy (Lu et al., 2020). In the present study, we found a strong association between hypoxia and the development of chemotherapy resistance. We then developed a model of top six genes most associated with drug resistance. Specifically, *DCBLD2*, a biomarker with largest risk to develop chemoresistance, was reported to promote CRC progression, and mediate drug resistance to 5-FU, a first-line drug for CRC (He et al., 2020; Xie et al., 2021). *PLEC*, a cytoskeletal gene, contributed to the invasiveness of neuroblastoma and resistance to cisplatin (Piskareva et al., 2015). *S100A11*, a member of calcium-binding proteins, promoted resistance of multiple cancers to 5-FU and cisplatin (Anania et al., 2013; Cui et al., 2021). Moreover, *PLAT* participated in proliferation, invasion and migration of non-small cell lung cancer as well as well as gefitinib resistance (Yamashita et al., 2015; Yan et al., 2020). *PPAP2B*, a lipid phosphate phosphohydrolase, promoted invasion of breast cancer (Westcott et al., 2015). *LAMC2* is a component of epithelial basement membrane shown to promote chemoresistance in several cancers (Moon et al., 2015; Liang et al., 2018; Okada et al., 2021).

The decision of postoperative chemotherapy for CRC patients has been based on clinicopathologic characteristics for several decades. Molecular biomarkers have great advantages in predicting disease outcome and thus may guide postoperative chemotherapy. Several commercial platforms, such as CRCassigner and ColoGuidePro, have been utilized in clinic for predicting patients’ prognosis and response to chemotherapy. Based on multiple and large cohorts, HGS shows its superiority in broad application and quantitative evaluation against the commercial platforms (Sveen et al., 2012; Borelli et al., 2021). Nevertheless, follow-up clinical trials need to be carried out to further verify the effectiveness of HGS.

## Conclusion

In summary, a novel gene signature based on 16 hypoxia-related genes was constructed, which could efficiently predict CRC prognosis. Besides, we found a strong association between hypoxia and stromal cells and immune status in the TME, which might be the underling mechanisms of hypoxia to determine CRC prognosis. Finally, we identified a list of six genes, which were highly correlated to drug resistance. HGS could be a useful tool in predicting CRC prognosis, as well as guiding personalized chemotherapy.

## Data Availability

Publicly available datasets were analyzed in this study. This data can be found here: GSE38832/GSE161158/GSE39582 https://www.ncbi.nlm.nih.gov/geo/query/acc.cgi TCGA http://xena.ucsc.edu/.

## Footnotes

1
https://gco.iarc.fr/today/home.

2
http://www.ncbi.nlm.nih.gov/geo.

3
http://xena.ucsc.edu/.

4
http://www.gsea-msigdb.org/gsea/msigdb/index.jsp.

5
https://cibersort.stanford.edu/.

6
http://bioinfo.life.hust.edu.cn/web/GSCALite.
