# Supplementary material for: The Expression Pattern of Hypoxia-Related Genes Predicts the Prognosis and Mediates Drug Resistance in Colorectal Cancer
Source: Front Cell Dev Biol. 2022 Jan 27;10:814621. doi: 10.3389/fcell.2022.814621 (PMC8829070; doi:10.3389/fcell.2022.814621)
Supplement: Supplementary file 1 [file DataSheet1.docx]

Supplementary Material

**
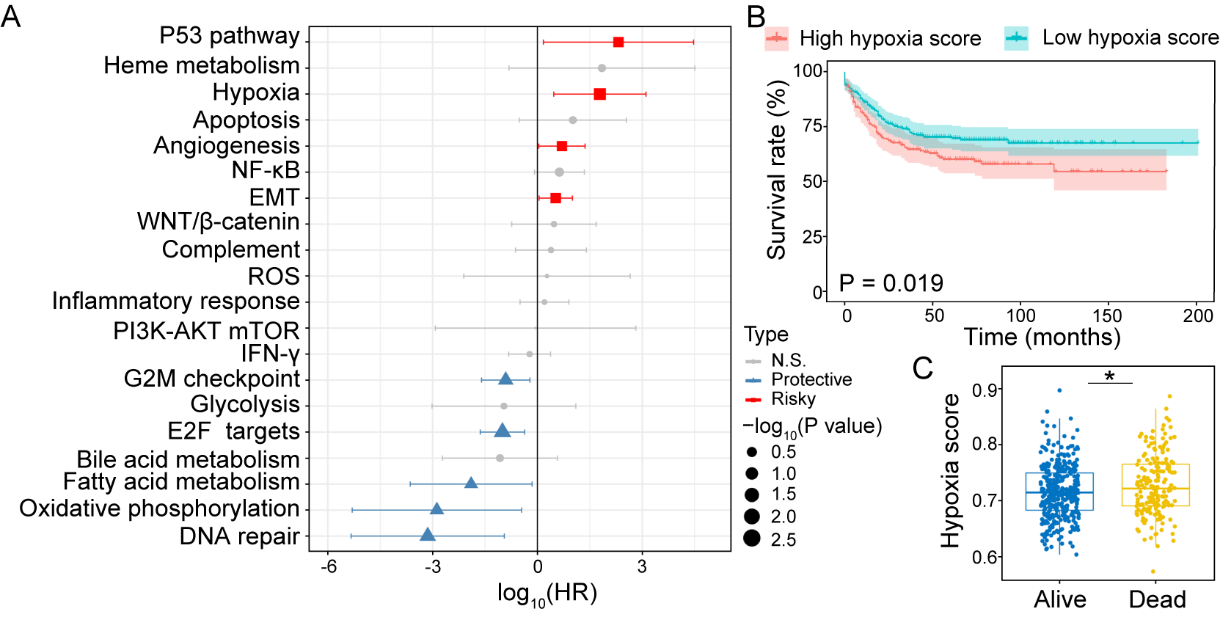
**

**Supplementary Figure S1.** Hypoxia is associated with CRC overall survival (OS). (A) Forest plot of hazard ratio (HR) for 20 prognostic cancer hallmarks. (B) Kaplan–Meier curves for patients with high hypoxia and low hypoxia scores. (C) Comparison of hypoxia scores in alive and dead patients.

**
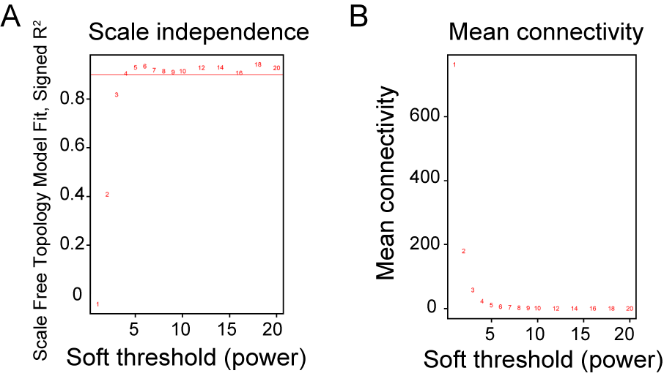
**

**Supplementary Figure S2.** Determination of the optimal soft-threshold power in WGCNA. (A) Calculation of the scale-free index for different soft-threshold powers (β). (B) Calculation of the mean connectivity for different soft-threshold powers.


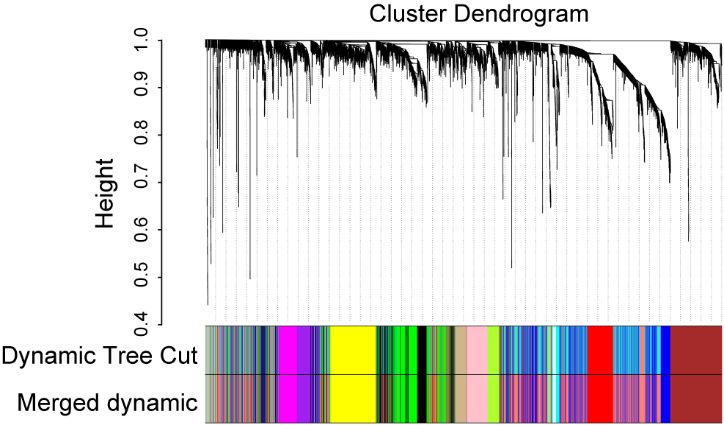


**Supplementary Figure S3.** Clustered dendrogram of differentially expressed genes.


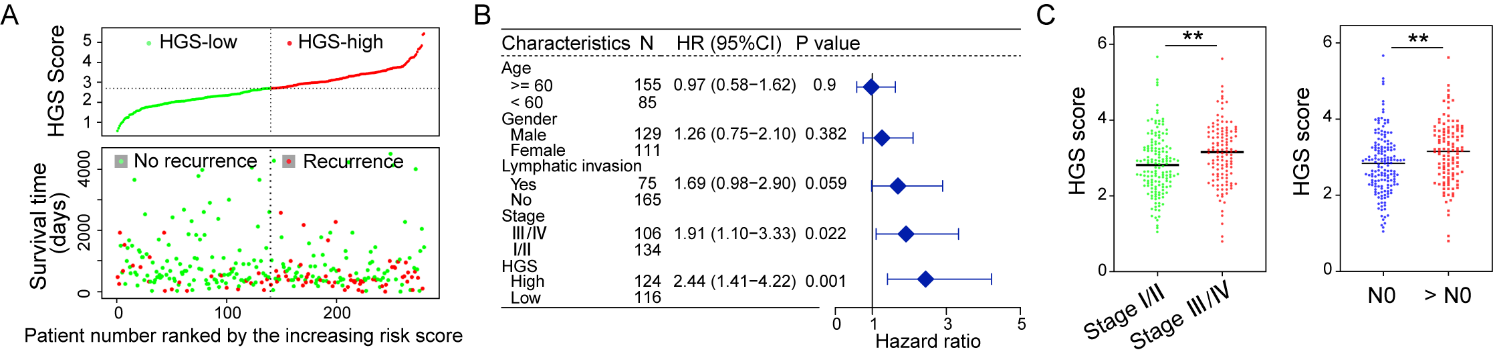


**Supplementary Figure S4.** Validation of HGS in TCGA. (A) The distribution of HGS score, patients’ status, and survival time. (B) Multivariate Cox regression analysis of HGS and clinicopathological characteristics. (C) Correlation of HGS with clinicopathological characteristics.


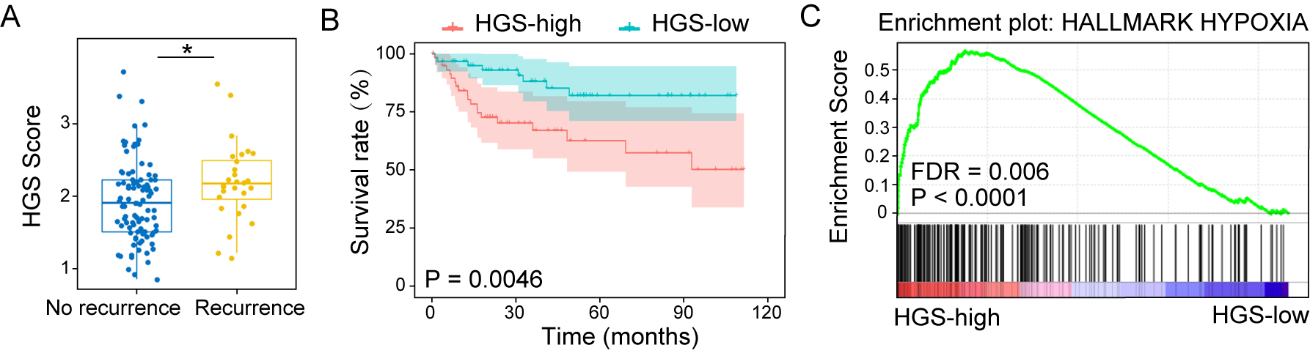


**Supplementary Figure S5.** Validation of HGS in GSE38832. (A) Comparison of HGS scores in recurrence and no recurrence patients. (B) Kaplan–Meier recurrence free survival (RFS) curves for patients in HGS-high and HGS-low groups. (C) GSEA of hypoxia pathway in HGS-high and HGS-low groups.


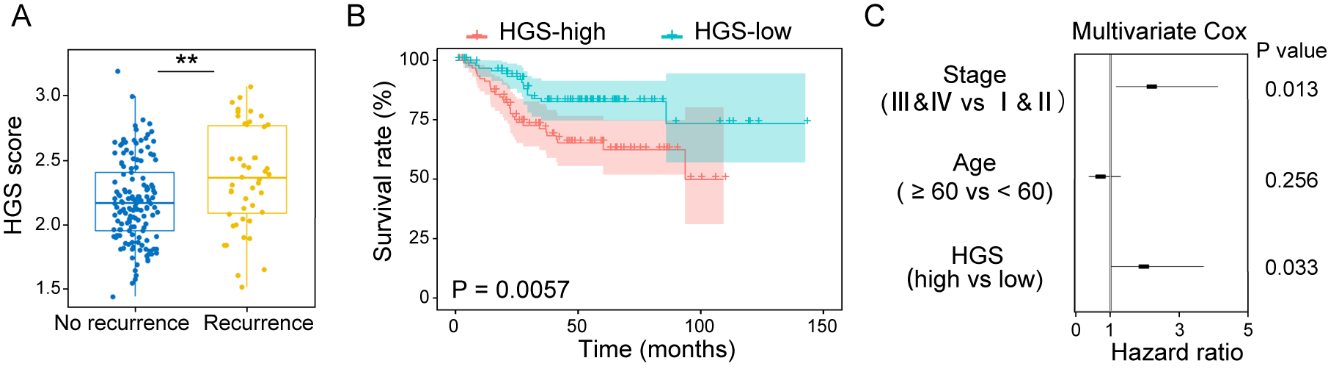


**Supplementary Figure S6.** Validation of HGS in GSE161158. (A) Comparison of HGS scores in recurrence and no recurrence patients. (B) Kaplan–Meier RFS curves for patients in HGS-high and HGS-low groups. (C) Multivariate Cox regression analysis of HGS and clinicopathological characteristics.


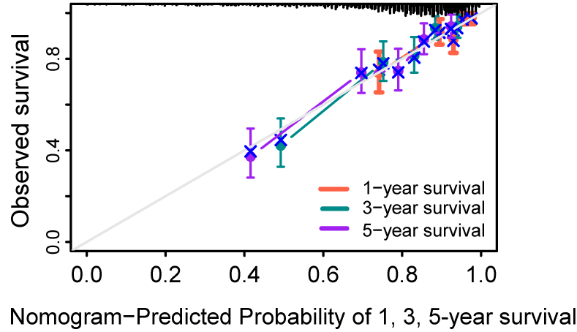


**Supplementary Figure S7.** Calibration analysis of the agreement between observed outcomes and nomogram predicted 1, 3, and 5-year survival.


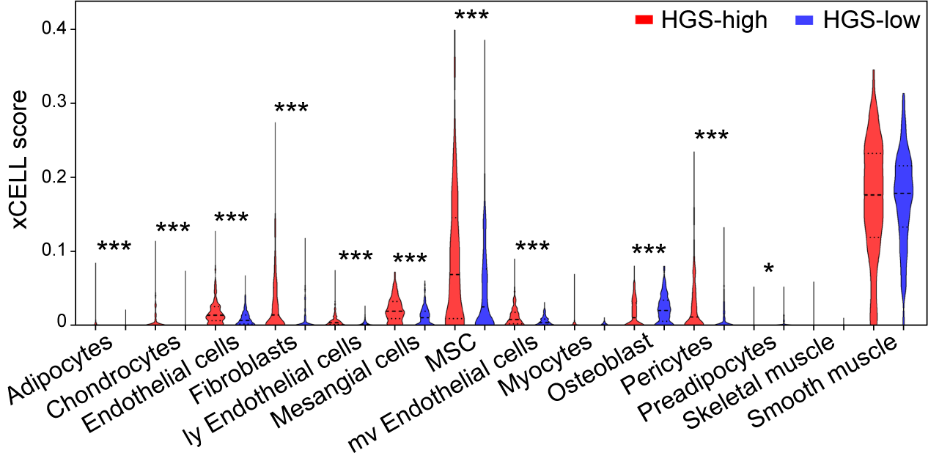


**Supplementary Figure S8.** The infiltration level of 14 stromal cells.


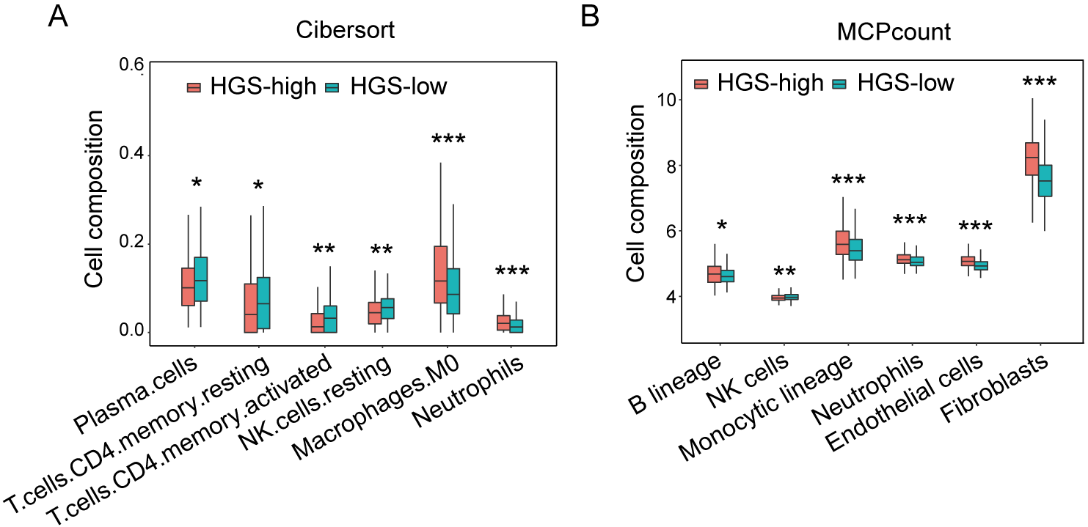


**Supplementary Figure S9.** Evaluation of immune cells infiltration using Cibersort (A) and MCPcount (B) in HGS-high and HGS-low groups.


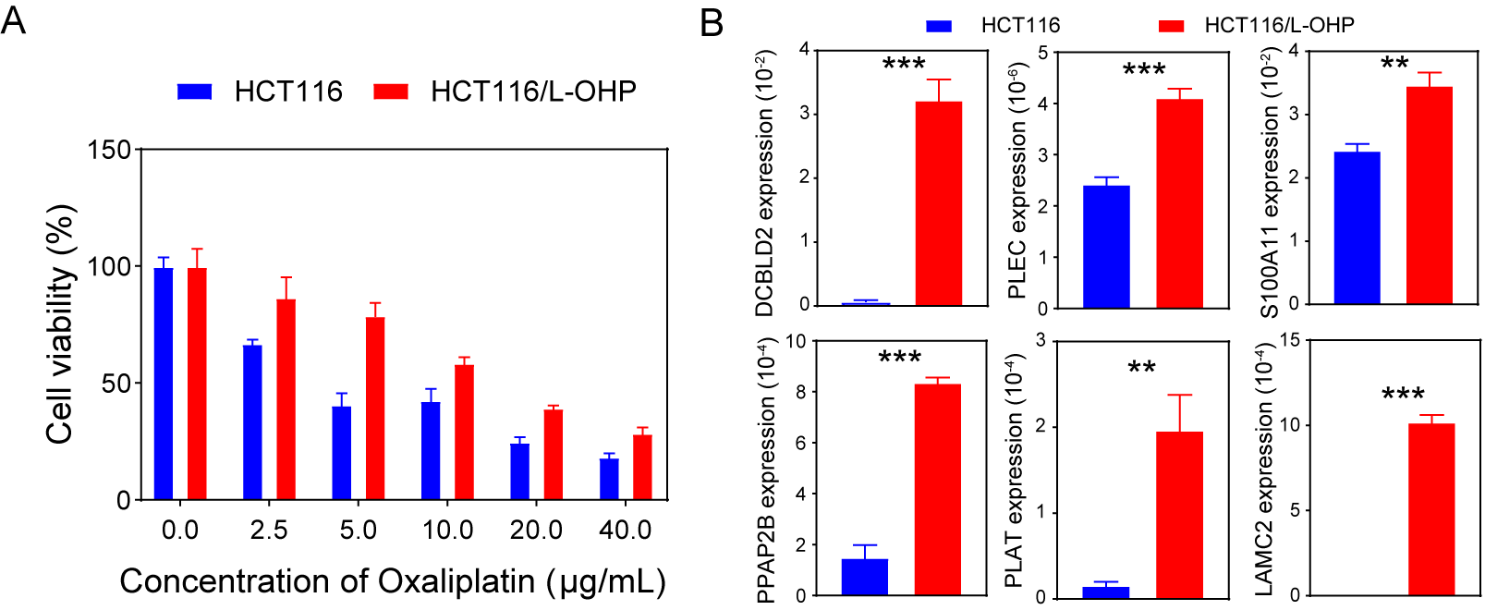


**Supplementary Figure S10.** (A) Cell viability of HCT116 and HCT116/L-OHP cells treated with different concentrations of oxaliplatin. (B) DCBLD2, PLEC, S100A11, PPAP2B, PLAT and LAMC2 mRNA level in HCT116 and HCT116/L-OHP cells.

**Supplementary Table S1.** Clinical characteristics of CRC patients from GSE39582, TCGA, GSE38832 and GSE161158 datasets.

|  | **GSE39582** | **TCGA** | **GSE38832** | **GSE161158** |
| --- | --- | --- | --- | --- |
| **All patients** | 518 | 279 | 122 | 191 |
| **Age** |  |  |  |  |
| **>=60** | 383 | 186 |  | 128 |
| **<60** | 135 | 93 |  | 63 |
| **Gender** |  |  |  |  |
| **Male** | 285 | 154 |  |  |
| **Female** | 233 | 125 |  |  |
| **Stage** |  |  |  |  |
| **Ⅰ** | 35 | 44 | 18 | 33 |
| **Ⅱ** | 253 | 108 | 35 | 74 |
| **Ⅲ** | 200 | 79 | 39 | 80 |
| **Ⅳ** | 30 | 39 | 30 | 4 |
| **Unknown** |  | 9 |  |  |
| **HGS** |  |  |  |  |
| **High** | 259 | 139 | 61 | 95 |
| **Low** | 259 | 140 | 61 | 96 |

**Supplementary Table S2.** Genes from drug resistant gene sets.

| **Drug resistant gene sets** | **Genes** |
| --- | --- |
| KANG_CISPLATIN_RESISTANCE  _UP | *ADIRF, AHNAK2, ATP7A, CDKN1A, DALRD3, EEF1AKMT3, FTH1, GRN, HSPA1B, IER5, IGFBP6, JUP, LZTFL1, MAFF, MDK, PCDHGC3, RAC2, SF3B3, TENT5A, TFPI, ZDHHC3* |
| GYORFFY_DOXORUBICIN  _RESISTANCE | *ABCB1, AC011525.2, ADAMTS1, ADD2, ANGPT1, AP4E1, BACE1, BBS12, BMP2, BMP7, BRWD1, CISH, CMPK1, CRYBG2, CST1, CYP27A1, FAAH, FAT4, FLJ38576, FMO2, FOXJ1, GJA5, HS3ST1, KRT40, LIMA1, MCPH1, NAV2, NSG2, P2RY6, PSG4, PTPRH, SLC38A2, SNTB1, STMN2, TIMP2, TRG-AS1, TXNDC17, TYMP, ZNF503* |
| KESHELAVA_MULTIPLE_DRUG  _RESISTANCE | *ACADS, ADAM15, ANXA2P2, BABAM2, BCAP31, BCL7B, BLVRB, C1orf35, C22orf46, CASP4, CCDC92, CCHCR1, CCS, CD151, CDK11B, CDKN2C, CRIP2, CYTH1, DNALI1, DOCK9, DXO, ENC1, FBXO11, FN1, FOXP3, GGA2, GSTK1, GUK1, H2BC20P, HDAC1, HFE, HGSNAT, HHAT, HSPB1, IBSP, ICAM3, IGHV5-51, INPP5B, IQGAP1, IZUMO4, KCTD17, KLRF1, LAMTOR2, LRRC14, AGOHB, MAN1B1, MAPK12, MARCHF2, MCUB, MEAK7, MED8, MSRB1, MYO1F, NXT2, OAS1, ODF2, PCTP, PDLIM5, PEX10, PHF1, PPIEL, PPP4C, PTHLH, RINT1, RPL23, SBNO2, SIL1, SLC25A13, SLC48A1, STYXL1, TAF12, TBX1, TEDC2, TMED10, TMEM104, TP53TG1, TSPAN31, TSPAN8, UFC1, USE1, VPS33B, ZNF668, ZSWIM8-AS1* |
| *TSAI_RESPONSE_TO_RADIATION*  *_THERAPY* | *A2M, BST2, CCN1, CCN2, CD69, CFB, COL4A1, COL6A1, COL6A2, COL6A3, EGR1, HBB, IFI6, IFIT2, IFITM1, IGFBP5, IL6, ISG15, LGALS3BP, LIPC, OAS3, PLAT, SERPINE1, SMAD3, TGFB2, TGFBR2, TGFBR3, THBS1, TNFRSF11B, TSC22D1, VEGFA, VEGFC* |

**Supplementary Table S3.** Sequences of primers.

| **Gene** | **Primer** | **Sequences (5’-3)** |
| --- | --- | --- |
| ***DCBLD2*** | F | TTGGTGGAAAGGAATGAAGC |
|  | R | CTGCAGCACTGTGGTGACTT |
| ***PLEC*** | F | CTTCCTCCCTGTCCCTTCC |
|  | R | AGGCCCACCTCAATCTCCT |
| ***S100A11*** | F | GAGTCCCTGATTGCTGTCTTC |
|  | R | AGGGTCCTTCTGGTTCTTTG |
| ***PLAT*** | F | GGTCTGGAGAAGTCTGTAGAG |
|  | R | CCTAGACTGGATTCGTGACAA |
| ***PPAP2B*** | F | CGTCCCGGAGAGCAAGAAC |
|  | R | AGGCCCGCCATGAAGAG |
| ***LAMC2*** | F | GCCTTTTGGCACCTGTATTC |
|  | R | CAGGATTCTCATCCCCTGAA |
| ***GAPDH*** | F | AAGGCTGTGGGCAAGG |
|  | R | TGGAGGAGTGGGTGTCG |
